# *S*tandardized *T*reatment *and* *D*iagnostic *A*pproach to *R*educe *D*isease burden in the early postoperative phase in children with congenital heart defects—STANDARD study: a pilot randomized controlled trial

**DOI:** 10.1007/s00431-023-05191-x

**Published:** 2023-09-21

**Authors:** Antonia Vogt, Sascha Meyer, Hans-Joachim Schäfers, Julius Johannes Weise, Stefan Wagenpfeil, Hashim Abdul-Khaliq, Martin Poryo

**Affiliations:** 1 Medical School, University of Saarland, Homburg/Saar, Germany; 2 Franz-Lust Klinik für Kinder- und Jugendmedizin, Städtisches Klinikum Karlsruhe, Karlsruhe, Germany; 3 Department of Thoracic and Cardiovascular Surgery, Saarland University Medical Center, Homburg/Saar, Germany; 4 Institute for Medical Biometry, Epidemiology and Medical Informatics, Saarland University Medical Center, Homburg/Saar, Germany; 5 Department of Pediatric Cardiology, Saarland University Medical Center, Kirrberger Straße, Building 9, D-66421 Homburg/Saar, Germany

**Keywords:** Congenital heart defect, Congenital heart surgery, Daily goals, Intensive care unit length of stay

## Abstract

To explore the effect of a daily goal checklist on pediatric cardiac intensive care unit (PCICU) length of stay (LOS) after congenital heart surgery. This study is a prospective randomized single-center study. Group characteristics were as follows: STANDARD group: *n* = 30, 36.7% female, median age 0.9 years; control group: *n* = 33, 36.4% female, median age 1.1 years. Invasive ventilation time, STAT categories, mean vasoactive-inotropic score (VIS)_24h_, maximal (max.) VIS_24h_, mean VIS_24–48h_, max. VIS_24–48h_, VIS category, number of sedatives, analgesics, diuretics, number of deployed diagnostic modalities, morbidities, and mortality did not differ between both groups. Median PCICU LOS was 96.0 h (STANDARD group) versus 101.5 h (control group) (*p* = 0.63). In the overall cohort, univariate regression analysis identified age at surgery (*b* = −0.02), STAT category (*b* = 18.3), severity of CHD (*b* = 40.6), mean VIS_24h_ (*b* = 3.5), max. VIS_24h_ (*b* = 2.2), mean VIS_24–48h_ (*b* = 6.5), and VIS category (*b* = 13.8) as significant parameters for prolonged PCICU LOS. In multivariate regression analysis, age at surgery (*b* = −0.2), severity of CHD (*b* = 44.0), and mean VIS_24h_ (*b* = 6.7) were of significance. Within the STANDARD sub-group, univariate regression analysis determined STAT category (*b* = 32.3), severity of CHD (*b* = 70.0), mean VIS_24h_ (*b* = 5.0), mean VIS_24–48h_ (*b* = 5.9), number of defined goals (*b* = 2.6), number of achieved goals (*b* = 3.3), number of not achieved goals (*b* = 10.8), and number of unevaluated goals (*b* = 7.0) as significant parameters for prolonged PCICU LOS. Multivariate regression analysis identified the number of defined goals (*b* = 2.5) and the number of unevaluated goals (*b* = −3.0) to be significant parameters.

*  Conclusion*: The structured realization and recording of daily goals is of advantage in patients following pediatric cardiac surgery by reducing PCICU LOS.

**Table:** 

**What is known:** *• Communication errors are the most frequent reasons for adverse events in intensive care unit patients.* *• Improved communication can be achieved by discussion and documentation of the patients’ goals during daily rounds.*	
**What is new:** *• In the overall cohort age at surgery, severity of congenital heart defect and mean vasoactive inotropic score within the first 24 hours had significant impact on pediatric cardiac intensive care unit (PCICU) length of stay (LOS).* *• In the intervention group, the number of defined goals and the number of unevaluated goals were significant parameters for prolonged PCICU LOS.*

**What is known:**

*• Communication errors are the most frequent reasons for adverse events in intensive care unit patients.*

*• Improved communication can be achieved by discussion and documentation of the patients’ goals during daily rounds.*

**What is new:**

*• In the overall cohort age at surgery, severity of congenital heart defect and mean vasoactive inotropic score within the first 24 hours had significant impact on pediatric cardiac intensive care unit (PCICU) length of stay (LOS).*

*• In the intervention group, the number of defined goals and the number of unevaluated goals were significant parameters for prolonged PCICU LOS.*

## Introduction

Congenital heart defects (CHD) are the most common congenital organ malformations of the newborn with a prevalence of about 8–14 per 1000 newborns [1, 2]. Worldwide, every year, approximately 1.35 million children are born with a CHD. Therefore, about 3 per 1000 newborns have a severe CHD [2, 3], often requiring congenital heart surgery or interventional procedures during the neonatal period.

Overall mortality in CHD patients declined significantly during the last decades. This can be attributed to improvements in diagnostics and therapy—among others, in congenital heart surgery procedures itself as well as in pre- and postoperative care at highly specialized pediatric cardiac intensive care units (PCICUs). Nonetheless, preterm infants, neonates, and infants under the age of 1 year with CHD remain a high-risk population for early mortality. Targeted measures to further improve their care are therefore urgently needed.

Treating critically ill children in intensive care units (ICUs) is becoming increasingly more complex. Working in multidisciplinary teams has been shown to be an effective approach to improve outcomes for this patient population [4–6]. However, in these teams, communication errors constitute a significant risk for various reasons. It is estimated that about 1.7 errors per patient and ICU day occur, with communication errors being the most frequent reason [5–7].

Thus, improving communication must be a top priority and can be achieved by discussion and documentation of the patients’ goals during daily rounds. Through the structured recording of these goals, the patients’ medical issues discussed can be transferred into plans of action [5]. Several study groups were able to show that this approach significantly improves the outcome of patients, advances communication between involved disciplines in multidisciplinary teams, and enhances the understanding of both caregivers and families regarding therapeutic plans made for the patients [7–10].

The aim of this prospective randomized single-center pilot study was to explore the effect of the implementation of a daily goals checklist on PCICU LOS of patients with CHD after congenital heart surgery procedures.

## Methods

### Study design and population

After institutional review board approval from the ethics committee of Saarland, Saarbrücken, Germany (file number 49/21), this prospective randomized single-center pilot study was performed at the tertiary PCICU of the Saarland University Medical Center, Homburg/Saar, Germany. The study was registered with Deutsches Register Klinische Studien (DRKS-ID: DRKS00025430).

**Fig. 1:**
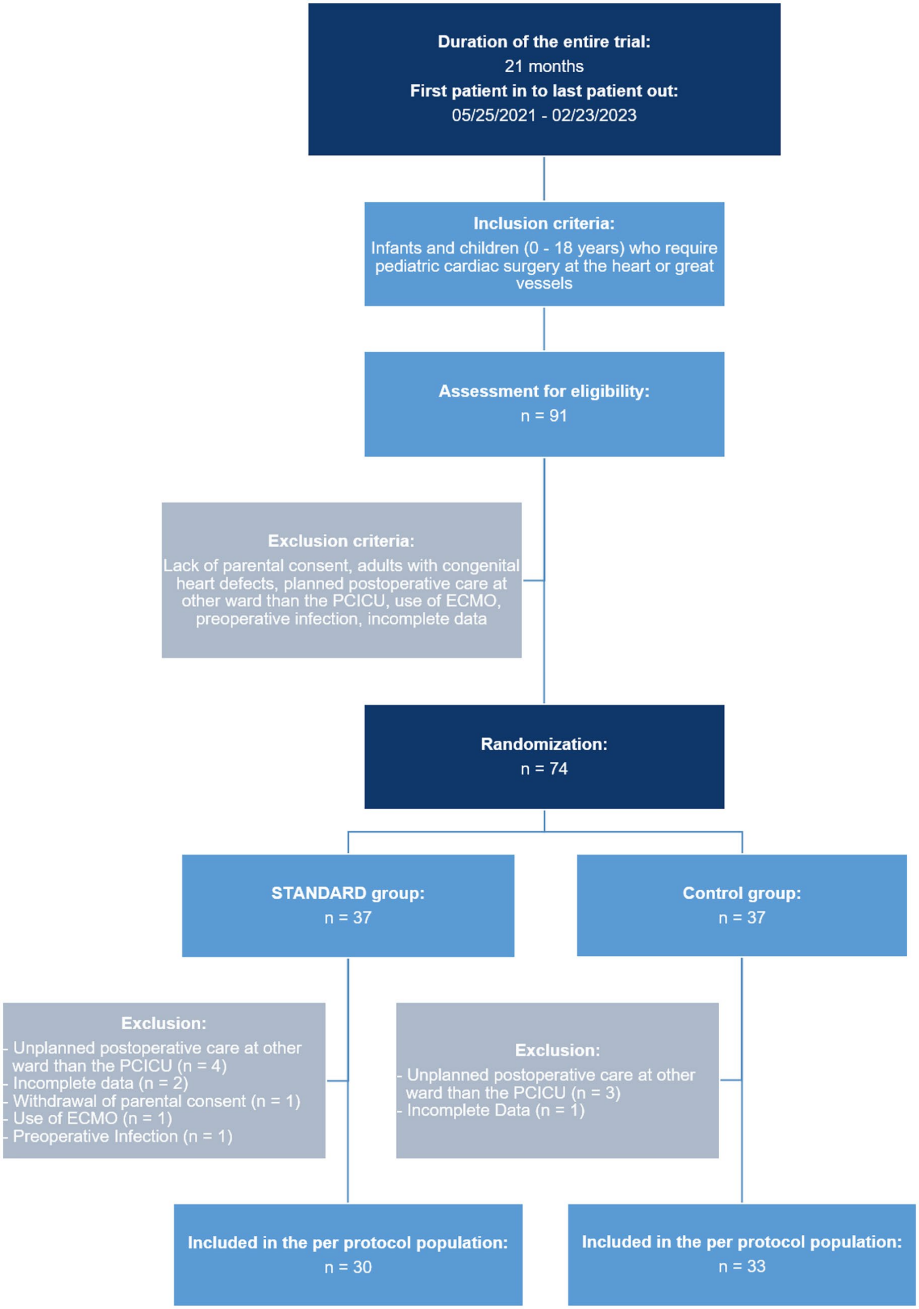
Trial design

During a period of 22 months (05/2021–02/2023), all patients aged 0–18 years with CHD, who required congenital heart surgery procedures and where parental consent was given, were included in this study (Fig. 1). Exclusion occurred in case of lack of parental consent, adults with CHD, postoperative care outside our PCICU, use of extracorporeal membrane oxygenation (ECMO), preoperative infection, and/or incomplete data (if missing data was in excess of 25%).

We generated a 1:1 randomization: group 1 = STANDARD group treated according to the STANDARD protocol and group 2 = control group treated conventionally. One person (MP) was responsible for the safekeeping of the randomization list. In case of a new recruitment, the responsible principal investigators (MP or SM) allotted the patient to one of the groups as mentioned above.

### Intervention

Included patients were randomized 1:1 into the intervention group (STANDARD group) and the control group.

**Fig. 2:**
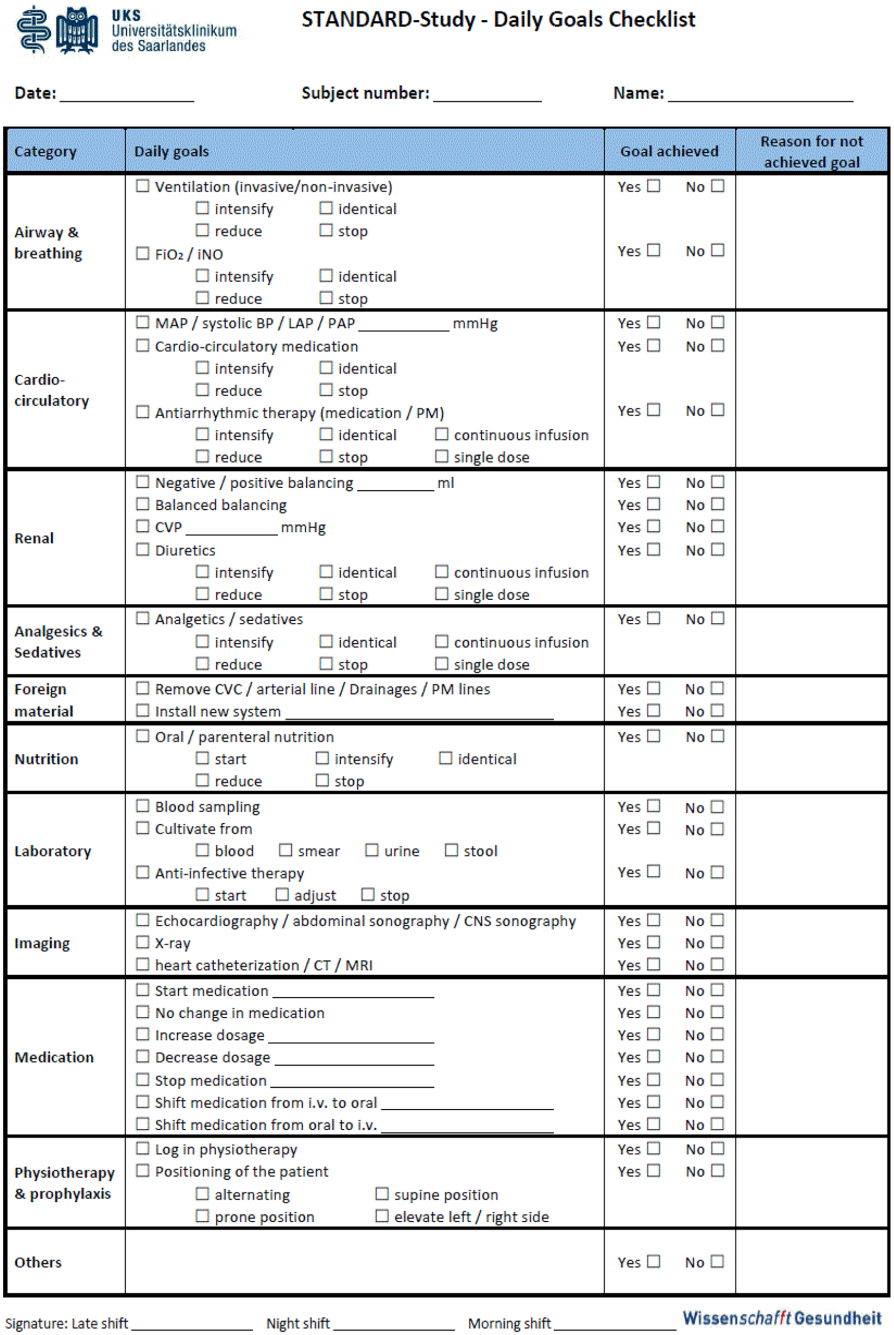
STANDARD protocol. BP blood pressure, CT computed tomography, CVC central venous catheter, CVP central venous pressure, FiO2 fraction of inspired oxygen, iNO inhaled nitric oxygen, i.v. intravenous, LAP left atrial pressure, MAP mean arterial pressure, MRI magnetic resonance tomography, PAP pulmonary arterial pressure, PM pacemaker

For the intervention group, we designed a special checklist (STANDARD protocol), which enabled the treating physician to define daily goals for each patient randomized into the STANDARD group (Fig. 2). The STANDARD protocol included 11 categories with 29 achievable daily goals. During morning rounds, the attending physician treating the patient evaluated whether the medical goals of the previous day had been achieved and set the goals to be achieved in the following 24 h. All physicians caring for this patient in each shift had to declare by signature that she/he had recognized the daily goals of the patient and had treated the patient accordingly. The STANDARD protocol was used during the entire PCICU stay. Defining daily goals was discontinued when the patient no longer required intensive care measures, regardless if transfer to the general pediatric cardiac unit was delayed due to over-occupancy.

The control group was treated according to our common practices in our PCICU.

### Primary outcome parameter

The primary outcome parameter was the length of stay at the pediatric cardiac intensive care unit (PCICU).

### Secondary outcome parameter

Invasive ventilation time, need for and dosage of catecholamines, need for and dosage of analgesics and sedatives, need for and dosage of diuretics, morbidities (infectious, cardio-circulatory, pulmonary, gastrointestinal, renal, central nervous system), mortality, and reimbursement were secondary outcome parameters.

### Definitions

The severity of CHDs was classified into mild, moderate, and severe according to previously published classifications [3, 11, 12].

The new 2020 Society of Thoracic Surgeons (STS)-European Association for Cardio-Thoracic Surgery (EACTS) Congenital Heart Surgery Mortality Score and Categories (STS-EACTS Mortality Score and Categories) or also commonly referred to as STAT Mortality Score and Categories were used to grade the congenital heart surgery procedures based on their risk for in-hospital mortality [13].

For quantification of the use of catecholamines, we employed the vasoactive-inotropic score (VIS). It includes the following vasoactive medications: dopamine, dobutamine, epinephrine, milrinone, vasopressin, and norepinephrine. The VIS was calculated by the formula previously published: dopamine dose (μg/kg/min) + dobutamine dose (μg/kg/min) + 100 × epinephrine dose (μg/kg/min) × (10 × milrinone dose (μg/kg/min) + 10 × vasopressin dose (mU/kg/min) + 100 × norepinephrine dose (μg/kg/min) [14]. We determined the maximal (max.) and mean VIS during the first 24 postoperative hours (VIS_24h_) as well as for the following 24–48 postoperative hours (VIS_24–48h_). Moreover, we grouped the patients into five categories (VIS categories 1–5) according to their VIS as previously published by Gaies et al. [15].

### Statistical analysis

For sample size planning, we assumed a reduction of PCICU LOS of 24 h. The determined group sample sizes were *n* = 64 for each group to achieve 80.146% power to reject the null hypothesis of equal means when the population mean difference is μ1 − μ2 = 7.0 − 6.0 = 1.0 (i.e., a mean reduction of the primary outcome PCICU LOS) with a standard deviation in the primary outcome for both groups of 2.0 and with a significance level (alpha) of 0.05 using a two-sided two-sample equal-variance *t*-test. Due to unforeseen decreased numbers of congenital heart surgery procedures in our institution, we reduced the number of study participants per group for this pilot study to *n* = 37.

Statistical analysis was made using IBM SPSS Statistics (IBM Corp. Released 2021. IBM SPSS Statistics for Macintosh, Version 29.0.0.0. Armonk, NY: IBM Corp). Data are presented as absolute numbers and percentages respectively median and range. For group comparisons of categorical data, the chi squared test was used. In case of cell frequencies < 5, Fisher’s exact test was employed. For statistical group comparisons of continuous data, we employed Student’s *t*-test for two independent groups. If the data deviated from normality assumptions, a Mann–Whitney *U* test was used for non-parametric statistical analysis. A two-sided *p*-value < 0.05 was considered statistically significant.

Univariate linear regression analysis was used to determine independent influential variables for a prolonged PCICU LOS. Results of linear regression analysis are depicted as a 95% confidence interval (95% CI) and regression coefficient B (*b*). In the second step, only significant parameters of the univariate linear regression analysis were included as independent variables in the multiple linear regression analysis. To avoid multicollinearity, in case of a Pearson correlation coefficient (*r*) > 0.7 between two variables, only one out of these two variables was included in further analysis.

## Results

### Study population

During the study period of 22 months, 91 patients were assessed for eligibility. After the application of exclusion criteria, 74 patients were randomized. Eleven patients were subsequently excluded because of unplanned postoperative care at another ward than our PCICU, incomplete data, withdrawal of parental consent, use of ECMO, and/or preoperative infection. Finally, 30 patients were included in the STANDARD group and 33 in the control group (Fig. 1).

Both groups did not differ with regard to sex (*p* = 0.98), age at surgery (*p* = 0.80), syndromic disease (*p* = 0.83), prematurity (*p* = 0.62), and severity of CHD (*p* = 0.69) (Table 1). There were 11/30 (36.7%) female patients in the STANDARD group and 12/33 (36.4%) in the control group. Median age at surgery was 0.9 years and 1.1 years (STANDARD group versus control group, *p* = 0.80), and there was no difference between the STAT categories (*p* = 0.52) (Tables 1 and 2). During PCICU stay, invasive ventilation time (*p* = 0.95), mean VIS_24h_ (*p* = 0.18), max. VIS_24h_ (*p* = 0.48), mean VIS_24–48h_ (*p* = 0.75), max. VIS_24–48h_ (*p* = 0.34), and VIS category (*p* = 0.13) as well as the number of sedatives (*p* = 0.99), analgesics (*p* = 0.46), and diuretics (*p* = 0.42) used did not differ significantly between both groups (Table 2). Also, the number of deployed diagnostic modalities and morbidities and mortality was not statistically different (*p* = 0.48) (Table 2). Median PCICU LOS in the STANDARD group was 96.0 h and 101.5 h in the control group (*p* = 0.63), while total hospital LOS was 12.6 days versus 14.1 days (*p* = 0.93) (Table 2).

**Table 1:** Patient’s characteristics

	**Overall cohort**			**Only mild CHDs**		
	**STANDARD group** **n = 30**	**Control group** **n = 33**	**p-value**	**STANDARD group** **n = 11**	**Control group** **n = 13**	**p-value**
**Gender**						
**male**	19 (63.3%)	21 (63.6%)	0.98^b^	7 (63.6%)	10 (76.9%)	0.66^b^
**female**	11 (36.7%)	12 (36.4%)		4 (36.4%)	3 (23.1%)	
**Age at surgery [years]**	median 0.9 (range 0.01 – 13.8)	median 1.1 (range 0.02 – 14.1)	0.80^d^	median 1.7 (range 0.3 – 11.5)	median 0.6 (range 0.3 – 11.4)	0.43^d^
**Syndromic disease**	8 (26.7%)	8 (24.2%)	0.83^b^	2 (18.2%)	3 (23.1%)	1.0^b^
**Tri21**	4 (13.3%)	4 (12.1%)		1 (9.1%)	3 (23.1%)	
**22q11**	1 (3.3%)	2 (6.1%)		0 (0.0%)	0 (0.0%)	
**Loeys-Dietz**	1 (3.3%)	1 (3.0%)		0 (0.0%)	0 (0.0%)	
**Marfan**	0 (0.0%)	1 (3.0%)		0 (0.0%)	0 (0.0%)	
**malformation syndrome**	2 (6.7%)	0 (0.0%)		1 (9.1%)	0 (0.0%)	
**Prematurity**	3 (10.0%)	3 (9.1%)	0.62^a^	0 (0.0%)	1 (7.7%)	1.0^a^
**Severity of CHD**			0.69^b^			n.a.
**Mild**	11 (36.7%)	13 (39.4%)		11 (100.0%)	13 (100.0%)	
**Small atrial septal defect (ASD)**	6 (54.5%)	4 (30.8%)		6 (54.5%)	4 (30.8%)	
**Small ventricular septal defect (VSD)**	3 (27.3%)	5 (38.5%)		3 (27.3%)	5 (38.5%)	
**ASD and VSD (combination, small)**	2 (18.2%)	3 (23.1%)		2 (18.2%)	3 (23.1%)	
**Isolated congenital aortic valve disease**	0 (0.0%)	1 (7.7%)		0 (0.0%)	1 (7.7%)	
**Moderate**	12 (40.0%)	10 (30.3%)				
**Coarctation of the aorta (ISTA)**	2 (16.7%)	1 (10.0%)				
**Partial anomalous pulmonary venous drainage (PAPVD)**	2 (16.7%)	1 (10.0%)				
**Patent ductus arteriosus (PDA)**	2 (16.7%)	2 (20.0%)				
**Right ventricular outflow tract obstruction (RVOTO)**	2 (16.7%)	0 (0.0%)				
**Aortic valve regurgitation**	1 (8.3%)	1 (10.0%)				
**Atrioventricular septal defect (AVSD)**	1 (8.3%)	1 (10.0%)				
**Mitral valve defect**	1 (8.3%)	0 (0.0%)				
**Tetralogy of Fallot (TOF)**	1 (8.3%)	1 (10.0%)				
**Aortopulmonary window (AP)**	0 (0.0%)	1 (10.0%)				
**ASD**	0 (0.0%)	1 (10.0%)				
**Coronary artery anomaly**	0 (0.0%)	1 (10.0%)				
**Severe**	7 (23.3%)	10 (30.3%)				
**Reconstruction of aortic arch (conduit)**	3 (42.9%)	4 (40.0%)				
**Double outlet right ventricle**	1 (14.3%)	2 (20.0%)				
**Hypoplastic left heart syndrome (HLHS)**	1 (14.3%)	0 (0.0%)				
**Pulmonary atresia**	1 (14.3%)	1 (10.0%)				
**Univentricular heart (UVH)**	1 (14.3%)	0 (0.0%)				
**Aortic stenosis (AS) (conduit, valved)**	0 (0.0%)	1 (10.0%)				
**Transposition of the great arteries (dTGA)**	0 (0.0%)	1 (10.0%)				
**Tricuspid atresia**	0 (0.0%)	1 (10.0%)				
**Congenital heart surgery procedures**						
**ASD repair**	6 (20.0%)	5 (15.2%)		6 (54.5%)	4 (30.8%)	
**VSD repair**	6 (20.0%)	5 (15.2%)		4 (36.4%)	5 (38.5%)	
**PA banding**	3 (10.0%)	1 (3.0%)		0 (0.0%)	0 (0.0%)	
**Coarctation repair**	2 (6.7%)	1 (3.0%)		0 (0.0%)	0 (0.0%)	
**PAPVD repair**	2 (6.7%)	1 (3.0%)		0 (0.0%)	0 (0.0%)	
**ASD and VSD repair**	1 (3.3%)	3 (9.1%)		1 (9.1%)	3 (23.1%)	
**ASD repair and PDA closure**	1 (3.3%)	1 (3.0%)		0 (0.0%)	0 (0.0%)	
**AVSD repair**	1 (3.3%)	1 (3.0%)		0 (0.0%)	0 (0.0%)	
**Interrupted aortic arch repair**	1 (3.3%)	1 (3.0%)		0 (0.0%)	0 (0.0%)	
**PDA closure**	1 (3.3%)	0 (0.0%)		0 (0.0%)	0 (0.0%)	
**Reconstruction of the aortic valve**	1 (3.3%)	2 (6.1%)		0 (0.0%)	1 (7.7%)	
**Reconstruction of the aortic valve and replacement of the ascending aorta**	1 (3.3%)	2 (6.1%)		0 (0.0%)	0 (0.0%)	
**Reconstruction of the mitral valve**	1 (3.3%)	0 (0.0%)		0 (0.0%)	0 (0.0%)	
**RVOTO procedure**	1 (3.3%)	0 (0.0%)		0 (0.0%)	0 (0.0%)	
**TOF repair**	1 (3.3%)	2 (6.1%)		0 (0.0%)	0 (0.0%)	
**Total cavopulmonary connection (Fontan)**	1 (3.3%)	3 (9.1%)		0 (0.0%)	0 (0.0%)	
**Anomalous aortic origin of coronary artery from aorta repair**	0 (0.0%)	1 (3.0%)		0 (0.0%)	0 (0.0%)	
**AP window repair and VSD repair**	0 (0.0%)	1 (3.0%)		0 (0.0%)	0 (0.0%)	
**Arterial switch procedure**	0 (0.0%)	1 (3.0%)		0 (0.0%)	0 (0.0%)	
**PA banding and PDA closure**	0 (0.0%)	1 (3.0%)		0 (0.0%)	0 (0.0%)	
**Ross procedure**	0 (0.0%)	1 (3.0%)		0 (0.0%)	0 (0.0%)	

Data are illustrated as absolute numbers and percentage respectively median and range

^a^Fisher's exact test if one of the expected cell frequencies was < 5

^b^Chi^2^ test if all the expected cell frequencies were ≥ 5

^c^T-test for comparison of means

^d^Mann-Whitney-U-Test

**Table 2:** Characteristics of cardiac surgery and pediatric cardiac intensive care unit stay of the overall group

	**Overall cohort**			**Only mild CHDs**		
	**STANDARD group** **n = 30**	**Control group** **n = 33**	**p-value**	**STANDARD group** **n = 11**	**Control group** **n = 13**	**p-value**
**Surgery**			0.31^b^			1.0^b^
**First surgery**	25 (83.3%)	24 (72.7%)		11 (100%)	12 (92.3%)	
**Re-surgery**	5 (16.7%)	9 (27.3%)		0 (0.0%)	1 (7.7%)	
**STAT category**			0.52^a^			n.a.
**1**	18 (60.0%)	24 (72.7%)		11 (100%)	13 (100%)	
**2**	5 (16.7%)	5 (15.2%)		0 (0.0%)	0 (0.0%)	
**3**	2 (6.7%)	0 (0.0%)		0 (0.0%)	0 (0.0%)	
**4**	4 (13.3%)	4 (12.1%)		0 (0.0%)	0 (0.0%)	
**5**	1 (3.3%)	0 (0.0%)		0 (0.0%)	0 (0.0%)	
**PCICU stay [hours]**	median 96.0 (range 48.0 – 360.0)	median 101.5 (range 43.0 – 309.0)	0.63^d^	median 72.0 (range 48.0 – 120.0)	median 95.5 (range 69.0 – 266.0)	0.02^d^
**Total length of hospital stay [days]**	median 12.6 (range 8.0 – 135.0)	median 14.1 (range 8.3 – 58.0)	0.93^d^	median 11.0 (range 8.0 – 22.1)	median 10.9 (range 8.3 – 18.2)	0.89^d^
**Invasive ventilation time [hours]**	median 15.3 (range 1.0 – 194.0)	median 12.0 (range 1.5 – 176.5)	0.95^d^	median 12.5 (range 2.0 – 43.0)	median 15.0 (range 1.5 – 70.5)	0.58^d^
**VIS**_**24h**_						
**Mean**	median 12.0 (range 3.6 – 29.7)	median 9.9 (range 2.0 – 29.1)	0.18^d^	median 10.6 (range 4.4 – 22.3)	median 10.2 (range 2.3 – 26.1)	0.54^d^
**Max.**	median 18.6 (range 4.4 – 43.1)	median 17.8 (range 2.6 – 68.1)	0.48^d^	median 16.0 (range 4.9 – 25.0)	median 14.2 (range 2.6 – 36.3)	0.64^d^
**VIS**_**24-48 h**_						
**Mean**	median 9.8 (range 1.8 – 35.3)	median 7.1 (range 0.1 – 14.4)	0.75^d^	median 4.2 (range 2.0 – 7.8)	median 7.7 (range 3.6 – 13.1)	0.045^d^
**Max.**	median 13.8 (range 3.0 – 40.3)	median 11.0 (range 1.1 – 31.2)	0.34^d^	median 11.4 (range 8.1 – 20.0)	median 11.4 (range 1.1 – 31.2)	
**VIS category**						
**1**	6 (20.0%)	14 (42.4%)	0.13^a^	2 (18.2%)	6 (46.2%)	0.13^a^
**2**	9 (30.0%)	3 (9.1%)		3 (27.3%)	1 (7.7%)	
**3**	4 (13.3%)	6 (18.2%)		1 (9.1%)	3 (23.1%)	
**4**	4 (13.3%)	2 (6.1%)		3 (27.3%)	0 (0.0%)	
**5**	7 (23.3%)	8 (24.2%)		2 (18.2%)	3 (23.1%)	
**Medication**						
**Sedatives**	median 2 (range 1 – 3)	median 2 (range 1 – 4)	0.99^d^	median 2 (range 1 – 2)	median 2 (range 1 – 4)	0.69^d^
**Analgesics**	median 2 (range 1 – 3)	median 2 (range 1 – 4)	0.46^d^	median 2 (range 2 – 2)	median 2 (range 1 – 3)	0.33^d^
**Diuretics**	median 1 (range 1 – 2)	median 1 (range 1 – 2)	0.42^d^	median 1 (range 1 – 2)	median 1 (range 1 – 2)	0.89^d^
**Diagnostics**						
**X-ray**	median 2 (range 1 – 9)	median 3 (range 1 – 9)	0.96^d^	median 2 (range 1 – 3)	median 2 (range 1 – 5)	0.72^d^
**Ultrasonography**	median 5 (range 1 – 17)	median 6 (range 2 – 16)	0.45^d^	median 4 (range 1 – 8)	median 5 (range 3 – 12)	0.19^d^
**Blood gas samples**	median 29 (range 12 – 103)	median 37 (range 12 – 91)	0.39^d^	median 24 (range 12 – 42)	median 30 (range 19 – 53)	0.02^d^
**lab**	median 7 (range 4 – 25)	median 8 (range 4 – 22)	0.52^d^	median 6 (range 4 – 10)	median 7 (range 5 – 15)	0.03^d^
**Total number of morbidities in**	18	33		5	13	
**n patients with morbidities**	12 (40%)]	20 (60.6%)	0.10^b^	3 (27.3%)	7 (53.8%)	0.24^b^
**Infectious**	0 (0.0%)	3 (9.1%)		0 (0.0%)	2 (15.4%)	
**Cardio-circulatory**	5 (27.8%)	9 (27.3%)		2 (40.0%)	5 (38.5%)	
**Pulmonary**	12 (66.7%)	18 (54.5%)		2 (40.0%)	5 (38.5%)	
**Gastrointestinal**	1 (5.6%)	3 (9.1%)		1 (20.0%)	1 (7.7%)	
**Renal**	0 (0.0%)	0 (0.0%)		0 (0.0%)	0 (0.0%)	
**Central nervous system**	0 (0.0%)	0 (0.0%)		0 (0.0%)	0 (0.0%)	
**Mortality**	1 (3.3%)	0 (0.0%)	0.48^a^	0 (0.0%)	0 (0.0%)	n.a.
**Reimbursement [Euros]**	median 26,650.7	median 30,243.7	0.36^c^	median 19,350.2	median 24,495.4	0.20^c^
	(range 15,853.2 – 254,330.0)	(range 16,570.1 – 107,866.6)		(range 15,853.2 – 47,665.4)	(range 16,570.1 – 43,490.3)	

Data are illustrated as absolute numbers and percentage respectively median and range

^a^Fisher's exact test if one of the expected cell frequencies was < 5

^b^Chi^2^ test if all the expected cell frequencies were ≥ 5

^c^T-test for comparison of means

^d^Mann-Whitney-U-Test

In the cohort of only mild CHDs, as in the overall cohort, there were no significant differences in the above-mentioned parameters except for mean VIS_24–48h_ (STANDARD group: median 4.2 versus control group: median 7.7, *p* = 0.045) and length of PCICU stay (STANDARD group: median 74.0 h versus control group: median 95.5 h, *p* = 0.02) (Tables 1 and 2).

### STANDARD protocol

In the STANDARD group, a median of 30.5 goals was defined per patient and PCICU stay, whereof a median of 25.0 (82.0%) goals was achieved (Table 3). With increasing severity of CHD, the number of defined goals during PCICU stay increased (mild CHD: median 23.0 goals, moderate CHD: 32.5 goals, severe CHD: 76.0 goals). Conversely, the proportion of achieved goals slightly decreased with increasing severity of CHD (mild CHD: 82.6%, moderate CHD: 78.5%, severe CHD: 75.0%). In all subgroups, about four-fifths of patients were treated according to the STANDARD protocol.

**Table 3:** Results of the goal-directed therapy

Total number of possible goals *n* = 29 per day of PCICU stay	**STANDARD group** **(overall)** ***n* = 30**	**STANDARD group** **(only mild CHDs)** ***n* = 11**	**STANDARD group** **(only moderate CHDs)** ***n* = 12**	**STANDARD group** **(only severe CHDs)** ***n* = 7**
Number of defined goals	Median 30.5 (range 15.0–134.0)	Median 23.0 (range 15.0–33.0)	Median 32.5 (range 17.0–134.0)	Median 76.0 (range 15.0–107.0)
Number of achieved goals	Median 25.0 (range 12.0–108.0)	Median 19.0 (range 13.0–28.0)	Median 25.5 (range 12.0–108.0)	Median 57.0 (range 14.0–101.0)
Number of unevaluated goals	Median 2.0 (range 0.0–21.0)	Median 2.0 (range 0.0–9.0)	Median 3.0 (range 0.0–11.0)	Median 4.0 (range 0.0–21.0)
Percentage of shifts not recognizing patient’s goals as per STANDARD protocol	18.8% ± 17.2%	23.1% ± 20.2%	18.1% ± 20.2%	13.1% ± 10.9%

### Factors associated with a prolonged PCICU stay

#### Overall cohort

Univariate linear regression analysis demonstrated that the following parameters were significantly associated with prolonged PCICU LOS in the overall cohort: age at surgery (*b* = −0.02), STAT category (*b* = 18.3), severity of CHD (*b* = 40.6), mean VIS_24h_ (*b* = 3.5), max. VIS_24h_ (*b* = 2.2), mean VIS_24–48h_ (*b* = 6.5), and VIS category (*b* = 13.8) (Table 4).

**Table 4:** Results of linear regression analysis—independent influential variables for PCICU stay in the overall cohort (STANDARD and control groups)

**Risk factor**	**Regression coefficient B**	**Standard error**	**Standardized coefficient Beta**	***t***	**Corrected *R***^**2**^	**95% confidence interval of *B***	***p*-value**
**Univariate linear regression**							
Age at surgery	−0.02	0.01	−0.3	−2.2	0.1	−0.03, −0.001	0.03
Syndromic disease	−6.5	23.3	−0.04	−0.3	0.001	−53.1, 40.1	0.78
STAT category	18.3	8.8	0.3	2.1	0.1	0.7, 35.9	0.04
Severity of CHD	40.6	11.6	0.4	3.5	0.2	17.4, 63.8	< 0.001
Mean VIS_24h_	3.5	1.3	0.3	2.7	0.1	0.9, 6.1	0.01
Max. VIS_24h_	2.2	0.8	0.3	2.7	0.1	0.6, 3.8	0.01
Mean VIS_24–48h_	6.5	1.6	0.6	4.1	0.3	3.3, 9.8	< 0.001
Max. VIS_24–48h_	2.5	1.5	0.3	1.7	0.04	−0.5, 5.5	0.10
VIS category	13.8	6.3	0.7	2.2	0.1	1.3, 26.3	0.03
Number of morbidities	16.2	16.3	0.2	1.0	0.00	−17.1, 49.5	0.33
**Multiple linear regression**							
Age at surgery	−0.2	0.01	−0.3	−2.4	n.a	−0.04, −0.003	0.02
STAT category	−9.1	10.4	−0.1	−0.9	n.a	−30.3, 12.1	0.39
Severity of CHD	44.0	14.3	0.5	3.1	n.a	14.9, 73.1	0.004
Mean VIS_24h_	−2.6	1.7	−0.3	−1.5	n.a	−6.1, 0.9	0.14
Mean VIS_24–48h_	6.7	2.0	0.6	3.4	n.a	2.6, 10.7	0.002

*n.a.* not applicable

Out of these significant parameters, the following had a Pearson correlation coefficient > 0.7: mean VIS_24h_ and max. VIS_24h_ (*r* = 0.75) and mean VIS_24h_ and VIS category (*r* = 0.83).

Finally, multiple linear regression analysis found the following parameters to be independently and significantly associated with prolonged PCICU LOS in the overall cohort: age at surgery (*b* = −0.2), severity of CHD (*b* = 44.0), and mean VIS_24h_ (*b* = 6.7) (Table 4).

#### STANDARD cohort

Within the STANDARD population, univariate linear regression analysis identified STAT category (*b* = 32.3), severity of CHD (*b* = 70.0), mean VIS_24h_ (*b* = 5.0), mean VIS_24–48h_ (*b* = 5.9), number of defined goals (*b* = 2.6), number of achieved goals (*b* = 3.3), number of not achieved goals (*b* = 10.8), and number of unevaluated goals (*b* = 7.0) as significant independent parameters for prolonged PCICU LOS (Table 5).

**Table 5:** Results of linear regression analysis— independent influential variables for PCICU stay in the STANDARD cohort

**Risk factor**	**Regression coefficient B**	**Standard error**	**Standardized coefficient Beta**	***t***	**Corrected *R***^**2**^	**95% confidence interval of *B***	***p*-value**
**Univariate linear regression**							
Age at surgery	−0.02	0.01	−0.3	−1.6	0.1	−0.04, 0.01	0.13
Syndromic disease	−33.8	35.7	−0.2	−0.9	−0.004	−106.9, 39.5	0.35
STAT category	32.3	11.7	0.5	2.8	0.2	8.3, 56.4	0.01
Severity of CHD	70.0	16.4	0.6	4.3	0.4	36.5, 103.5	< 0.001
Mean VIS_24h_	5.0	2.1	0.4	2.5	0.2	0.8, 9.3	0.02
Max. VIS_24h_	2.6	1.7	0.3	1.5	0.1	−0.9, 6.1	0.14
Mean VIS_24–48h_	5.9	2.0	0.6	2.9	0.3	1.6, 10.3	0.01
Max. VIS_24–48h_	3.4	2.3	0.4	1.5	0.1	−1.3, 8.2	0.16
VIS category	14.1	10.6	0.2	1.3	0.03	−7.6, 35.8	0.19
Number of morbidities overall	30.4	18.7	0.5	1.6	0.1	−11.3, 72.2	0.14
Number of defined goals	2.6	0.2	0.9	15.2	0.9	2.3, 3.0	< 0.001
Number of achieved goals	3.3	0.2	1.0	15.5	0.9	2.8, 3.7	< 0.001
Number of not achieved goals	10.8	1.9	0.7	5.5	0.5	6.8, 14.7	< 0.001
Number of unevaluated goals	7.0	3.2	0.4	2.2	0.1	0.4, 13.6	0.04
Percentage of shifts not recognizing patient’s goals as per STANDARD protocol	8.3	95.0	0.02	0.1	0.9	−186.3, 202.9	0.93
**Multiple linear regression**							
Severity of CHD	18.7	9.7	0.2	1.9	n.a	−2.7, 40.1	0.08
Mean VIS_24h_	−0.9	1.0	−0.1	−0.9	n.a	−3.2, 1.3	0.38
Mean VIS_24–48h_	0.1	1.1	0.02	0.1	n.a	−2.2, 2.5	0.89
Number of defined goals	2.5	0.3	0.9	9.1	n.a	1.9, 3.1	< 0.001
Number of unevaluated goals	−3.0	1.2	−0.2	−2.6	n.a	−5.5, −0.5	0.03

Pearson’s correlation coefficient was *r* = 0.75 for the STAT category and severity of CHD, *r* = 0.96 for the number of defined goals and the number of achieved goals respectively *r* = 0.83 for the number of defined goals and the number of not achieved goals.

Multiple linear regression analysis identified the number of defined goals (*b* = 2.5) and number of unevaluated goals (*b* = −3.0) as significant parameters for prolonged PCICU LOS (Table 5).

#### Mild congenital heart defects

In the cohort of mild CHDs—STANDARD group plus control group—univariate linear regression analysis found no significant parameters (Table 6). However, in the STANDARD group with only mild CHDs, the number of defined goals (*b* = 3.2) and the number of achieved goals (*b* = 4.0) had a significant impact on PCICU stay LOS (Table 7). Since these two variables had a Pearson’s correlation coefficient of *r* = 0.90, no multiple linear regression analysis was performed.

**Table 6:** Results of the univariate linear regression—independent influential variables for PCICU stay for all patients (STANDARD and control groups) with mild congenital heart defects

**Risk factor**	**Regression coefficient B**	**Standard error**	**Standardized coefficient Beta**	**t**	**Corrected R**^**2**^	**95%—confidence interval of B**	**p-value**
Age at surgery	0.01	0.01	0.1	0.7	−0.03	−0.01, 0.03	0.52
Syndromic disease	−32.3	29.2	−0.2	−1.1	0.01	−92.8, 28.2	0.28
Mean VIS_24h_	−0.8	1.9	−0.1	−0.4	−0.04	−4.83, 3.2	0.67
Max. VIS_24h_	−0.9	1.3	−0.1	−0.7	−0.03	−3.6, 1.9	0.51
Mean VIS_24–48h_	1.8	4.2	0.1	0.4	−0.1	−7.3, 11.0	0.67
Max. VIS_24–48h_	−3.1	2.3	−0.3	−1.4	0.1	−8.0, 1.8	0.19
VIS category	−4.0	7.9	−0.1	−0.5	−0.03	−20.3, 12.3	0.62
Number of morbidities	−16.8	26.9	−0.2	−0.6	−0.1	−78.9, 45.3	0.55

**Table 7:** Results of the univariate linear regression—independent influential variables for PCICU stay for patients within the STANDARD cohort with mild congenital heart defects

**Risk factor**	**Regression coefficient B**	**Standard error**	**Standardized coefficient Beta**	***t***	**Corrected *R***^**2**^	**95%—confidence interval of *B***	***p*-value**
Age at surgery	−0.01	0.01	−0.4	−1.2	0.1	−0.02, 0.01	0.27
Syndromic disease	12.0	18.2	−0.2	0.7	−0.1	−29.3, 53.3	0.53
Mean VIS_24h_	1.9	1.2	0.5	1.6	0.1	−0.8, 4.5	0.15
Max. VIS_24h_	0.8	0.9	0.3	0.9	0.01	−1.3, 2.9	0.41
Mean VIS_24–48h_	−4.4	5.3	0.4	0.8	−0.1	−19.1, 10.4	0.46
Max. VIS_24–48h_	−3.2	2.1	−0.6	−1.5	0.4	−9.0, 2.6	0.20
VIS category	4.4	4.9	0.3	0.9	0.1	−6.7, 15.4	0.39
Number of morbidities	12.0	62.4	0.2	0.2	−0.9	−780.3, 804.3	0.88
Number of defined goals	3.2	0.8	0.8	3.9	0.6	1.4, 5.1	0.004
Number of achieved goals	4.0	0.9	0.8	4.3	0.6	1.9, 6.2	0.002
Number of not achieved goals	1.0	5.6	0.1	0.2	0.9	−11.7, 13.6	0.87
Percentage of shifts not recognizing patient’s goals as per STANDARD protocol	52.6	33.1	0.5	1.6	0.1	−22.3, 127.4	0.15

### Drug dosage

The dosage of the most common used medications applied via continuous intravenous infusion other than vasoactive medication did not differ between the STANDARD group and the control group: morphine (median 0.0 µg/kg/h, range 0.0–31.8 µg/kg/h versus median 0.0 µg/kg/h, range 0.0–16.8 µg/kg/h; *p* = 0.38), piritramide (median 0.6 mg/kg/d, range 0.0–1.4 mg/kg/d versus median 0.8 mg/kg/d, range 0.0–2.7 mg/kg/d; *p* = 0.16), metamizole (median 50.8 mg/kg/d, range 0.0–60.2 mg/kg/d versus median 51.0 mg/kg/d, range 0.0–60 mg/kg/d; *p* = 0.90), clonidine (median 0.6 µg/kg/h, range 0.0–1.2 µg/kg/h versus median 0.6 µg/kg/h, range 0.0–1.8 µg/kg/h; *p* = 0.36), midazolam (median 0.1 µg/kg/h, range 0.0–0.3 mg/kg/h versus median 0.1 mg/kg/h, range 0.0–0.2 mg/kg/h; *p* = 0.72), furosemide (median 0.4 mg/kg/h, range 0.0–0.5 mg/kg/h versus median 0.4 mg/kg/h, range 0.0–0.6 mg/kg/h; *p* = 0.85), and ethacrynic acid (median 0.0 mg/kg/h, range 0.0–0.1 mg/kg/h versus median 0.0 mg/kg/h, range 0.0–0.1 mg/kg/h; *p* = 0.58).

## Discussion

In this prospective randomized single-center pilot study, we explored the effect of the implementation of a daily goals checklist on PCICU LOS in pediatric patients with CHD after congenital heart surgery procedures. In the overall cohort, we were able to detect a slightly but non-significantly reduced PCICU LOS in the STANDARD group (median 96.0 h) versus the control group (median 101.5 h) (*p* = 0.63). However, in the cohort of mild CHDs, we found a significantly reduced PCICU LOS in the STANDARD group of only 74.0 h versus 95.5 h in the control group (*p* = 0.02).

In 2003, Pronovost et al. [8] evaluated for the first time the effectiveness of standardized communication in the setting of an ICU by using daily goals forms. They found, that in their participating ICU before the intervention, only about 10% of caregivers understood the patients’ daily goals. In contrast, after the implementation of the daily goals form, more than 95% were aware of the patients’ daily goals. Moreover, they found a decreased ICU LOS by 1 day (2.2 days versus 1.1. days) after implementation of the daily goals form. Contrary, Roy and colleagues [16] found no difference in ICU LOS and total hospital LOS in their investigation. Among others, they evaluated the outcomes after the implementation of an enhanced recovery program in congenital cardiac surgery and found increased extubation rates in the operating room, decreased ventilation times, and reduced need for opioids. This is contrary to our findings where no differences between the intervention and the control groups related to ventilation time and drug dosage could be demonstrated. However, in line with our findings, Roy et al. [16] found shorter ICU LOS and total hospital LOS for lower-risk surgical procedures.

The reason for the different findings in our study regarding PCICU LOS in the overall cohort and in patients with mild CHDs remains to be elucidated. We speculate that in complex congenital heart surgery procedures, postoperative care is more challenging and results in increased bedside presence of treating physicians even without the definition of daily goals. As a consequence, pathologic parameters are recognized, and treatment is initiated more readily compared to patients with less complex CHD. Therefore, patients with mild CHD could benefit from a daily goals checklist, which would increase bedside presence and more compelling therapy. Pronovost and colleagues [17] examined ICU physician staffing and found high-intensity physician staffing to be associated with reduced ICU LOS and hospital LOS. In our analysis, the PCICU LOS and the total hospital LOS are slightly reduced in the STANDARD group. However, in contrast, in the STANDARD cohort of mild CHDs, there is only a significant reduction of PCICU LOS but not in total hospital LOS. In general, a shorter PCICU LOS is expected to also result in a shorter total hospital LOS [18]. However, the limited number of study participants might also contribute to the different findings on PCICU LOS since sample size planning calculated a higher case number per group to detect significant differences.

Our analysis identified several significant parameters for prolonged PCICU LOS. Multiple linear regression analysis found age at surgery (*b* = −0.2), severity of CHD (*b* = 44.0), and mean VIS_24h_ (*b* = 6.7) to be significant contributors to PCICU LOS in the overall cohort. In the sub-analysis of the STANDARD group, however, these parameters were only in part significantly associated with PCICU LOS in the univariate linear regression analysis, while multiple linear regression analysis only identified number of defined goals (*b* = 2.5) and number of unevaluated goals (*b* = −3.0) as relevant influential variables for prolonged PCICU LOS. Similar findings were also apparent in the STANDARD group with mild CHDs with the number of defined goals (*b* = 3.2) and the number of achieved goals (*b* = 4.0) as significant influential variables, whereas in the overall cohort of patients with mild CHDs, no significant parameters were determined.

Age at surgery is an important contributor to PCICU LOS. Alexander and colleagues [19] designed a prediction tool for patients who will remain in the PCICU for more than 7 days after the congenital heart surgery procedure and found age at surgery, mechanical ventilation, and admission status at the time of surgery to be predictors of the outcome measure. Brown et al. [20] showed that neonates carry a higher risk for prolonged PCICU LOS.

Besides age at surgery, severity of CHD was also associated with a prolonged PCICU LOS in multiple linear regression analysis in the present study. Although severity of CHD was not associated with the STAT category in the overall cohort, in the sub-analysis of the STANDARD, group both variables were associated with each other (*r* = 0.75), and STAT categories were independent influential variables for prolonged PCICU LOS in the univariate linear regression analysis in both the overall cohort and in the STANDARD group (*b* = 18.3 respectively *b* = 32.3). Gillespie et al. [21] found that a higher Aristotle Basic Complexity Score increased PCICU LOS. The present study confirms this. Although STAT categories and Aristotle Basic Complexity Scores are not identical, they are somewhat comparable: Kogon and Oster [22] found areas under the receiver operating characteristic (ROC) curves of 0.76 and 0.82 for the STAT categories and Aristotle Basic Complexity Score for prolonged hospital stay, and Cavalcanti and colleagues [23] could also demonstrate that both scores had almost the same areas under ROC curve concerning mortality outcome (0.74 versus 0.77).

The VIS is an often-examined tool to predict morbidity and mortality [14, 15, 24–27]. Among others, McIntosh et al. [14] and Davidson et al. [26] found an association of the VIS and the length of pediatric ICU stay. In their studies, VIS at 48 h was significantly associated with both ventilator days and ICU LOS. However, they did not calculate mean VIS for different time intervals but VIS at certain points in time which might explain why we were able to find mean VIS_24h_ to be significant independent influential variables for prolonged PCICU LOS.

In the STANDARD cohort, the above-discussed parameters were not of relevance as significant parameters for prolonged PCICU LOS. Here, the number of defined goals (*b* = 2.5) and the number of unevaluated goals (*b* = −3.0) were relevant influential parameters for prolonged PCICU LOS respectively, and the number of defined goals (b = 3.2) and the number of achieved goals (b = 4.0) in the sub-group of STANDARD patients were relevant influential parameters with only mild CHDs. These findings underline the importance of these parameters for PCICU LOS. The explanation for the increase of PCICU LOS in case of increasing numbers of defined goals might be explained by the severity of CHDs: in mild CHDs, the number of defined goals was in a median of 23.0, in moderate CHDs in a median of 32.5, and in severe CHDs in a median of 76.0. Thus, the increasing number of defined goals depicts the severity of CHD, and as described previously, there was a high correlation between the severity of CHD and the STAT category (*r* = 0.75) in our cohort. In turn, increasing STAT categories were associated with prolonged PCICU LOS. The regression coefficient B of *b* = −0.3 for the number of unevaluated goals in the multiple linear regression analysis is contrary to *b* = 7.0 in univariate linear regression analysis. This is explained by so-called suppression variables.

The effect of daily goals on drug dosages is unclear. In our cohort, there was no difference between the STANDARD group and the control group with regard to mean and max. VIS, dosage of opioids, analgesics, sedatives, and diuretics administered via continuous intravenous infusion. To the best of our knowledge, there are no surveys which examine the effect of daily goals on the drug dosages used to achieve the defined goals. However, literature on goal-directed therapy reports inconsistent data; in some cases, there is no difference between the intervention group and the control group; in others, there are significant differences [28–34].

### Limitations

This survey has some shortcomings. Due to the decreased number of congenital heart surgery procedures in our institution for different reasons, we had to reduce the number of study participants to complete the trial. In doing so, it is possible that differences between studied groups were not detected as significant. However, as a pilot study, this trial has the potential to provide a basis for further studies.

Treating physicians were able to choose a vast number of daily goals which makes it difficult to fully assess the relevance of the different daily goals. Moreover, no specific instructions (e.g., how to reach a negative fluid balance of > −500 ml) per daily goals were made because of the great variability between the treated patients. Conversely, our STANDARD protocol represents the need for individualized treatment and meets the requirements of pediatric patients.

It is important to stress that in about one-fifth of medical shifts, the patients were not treated as per STANDARD protocol. This might have an impact on our results. As this was almost identical between different subgroups, data are still comparable.

## Conclusion

We were able to identify several significant parameters for prolonged PCICU LOS. The fact that in our STANDARD group, only parameters of the STANDARD protocol like the number of defined goals, number of achieved goals, and number of unevaluated goals had a significant impact on PCICU LOS but not general parameters, like age at surgery, severity of CHD, or mean VIS_24h_ as seen in the overall cohort, stresses the relevance of the daily goals checklist. Although daily goals are frequently orally discussed during daily rounds, the structured implementation and realization of daily goals in written form appears to be of advantage for patients in the setting of PCICU.

## Data Availability

Upon personal request, we will provide readers with the data used in this publication.

## Abbreviations

95%-CI: 95%-Confidence interval
*b*: Regression coefficient B
CHD: Congenital heart defects
DRKS: Deutsches Register Klinische Studien
EACTS: European Association for Cardio-Thoracic Surgery
ECMO: Extracorporeal membrane oxygenation
GDT: Goal-directed therapy
ICU: Intensive care unit
LOS: Length of stay
*r*: Pearson’s correlation coefficient
STS: Society of Thoracic Surgeons
PCICU: Pediatric cardiac intensive care units
VIS: Vasoactive-inotropic score
